# Treatment-related amenorrhea in a modern, prospective cohort study of young women with breast cancer

**DOI:** 10.1038/s41523-021-00307-8

**Published:** 2021-07-27

**Authors:** Philip D. Poorvu, Jiani Hu, Yue Zheng, Shari I. Gelber, Kathryn J. Ruddy, Rulla M. Tamimi, Jeffrey M. Peppercorn, Lidia Schapira, Virginia F. Borges, Steven E. Come, Ellen Warner, Matteo Lambertini, Shoshana M. Rosenberg, Ann H. Partridge

**Affiliations:** 1 Dana-Farber Cancer Institute, Boston, MA USA; 2 Mayo Clinic, Rochester, MN USA; 3 Weill Cornell Medicine, New York, NY USA; 4 Massachusetts General Hospital, Boston, MA USA; 5 Stanford University, Palo Alto, CA USA; 6 University of Colorado Cancer Center, Aurora, CO USA; 7 Beth Israel Deaconess Medical Center, Boston, MA USA; 8 Sunnybrook Health Sciences Centre, Toronto, ON Canada; 9 University of Genova, Genova, Italy; 10 IRCCS Ospedale Policlinico San Martino, Genova, Italy

**Keywords:** Breast cancer, Breast cancer

## Abstract

Young women with breast cancer experience unique treatment and survivorship issues centering on treatment-related amenorrhea (TRA), including fertility preservation and management of ovarian function as endocrine therapy. The Young Women’s Breast Cancer Study (YWS) is a multi-center, prospective cohort study of women diagnosed at age ≤40, enrolled from 2006 to 2016. Menstrual outcomes were self-reported on serial surveys. We evaluated factors associated with TRA using logistic regression. One year post-diagnosis, 286/789 (36.2%) experienced TRA, yet most resumed menses (2-year TRA: 120/699; 17.2%). Features associated with 1-year TRA included older age (OR_≤30vs36-40 _= 0.29 (0.17–0.48), OR_31-35vs36-40 _= 0.67 (0.46–0.94), *p* = 0.02); normal body mass index (BMI) (OR_≥25vs18.5-24._ =0.59 (0.41–0.83), *p* < 0.01); chemotherapy (OR_chemo vs no chemo_ = 5.55 (3_._60–8.82), *p* < 0.01); and tamoxifen (OR = 1.55 (1.11–2.16), *p* = 0.01). TRA rates were similar across most standard regimens (docetaxel/carboplatin/trastuzumab +/− pertuzumab: 55.6%; docetaxel/cyclophosphamide +/− trastuzumab/pertuzumab: 41.8%; doxorubicin/cyclophosphamide/paclitaxel +/− trastuzumab/pertuzumab: 44.1%; but numerically lower with AC alone (25%) or paclitaxel/trastuzumab (11.1%). Among young women with breast cancer, lower BMI appears to be an independent predictor of TRA. This finding has important implications for interpretation of prior studies, future research, and patient care in our increasingly obese population. Additionally, these data describe TRA associated with use of docetaxel/cyclophosphamide, which is increasingly being used in lieu of anthracycline-containing regimens. Collectively, these data can be used to inform use of fertility preservation strategies for women who need to undergo treatment as well as the potential need for ovarian suppression following modern chemotherapy for young women with estrogen-receptor-positive breast cancer.

**Clinical trial registration:** www.clinicaltrials.gov, NCT01468246.

## Introduction

Young women with breast cancer, defined as those diagnosed at age ≤40 years, represent a small minority of breast cancer patients overall^1^. However, the optimization of breast cancer outcomes and breast cancer survivorship for young women requires attention to several unique issues, many of which center around ovarian function at and following diagnosis^2^. Young age has historically been considered a risk factor for disease recurrence^3,4^ and recent data have demonstrated that this risk appears to be predominantly among those with luminal A and luminal B breast cancer subtypes^5^. To what extent this risk stems from the biology of premenopausal breast cancer versus suboptimal endocrine therapy remains poorly understood. Ovarian function suppression (OFS) is a well-recognized therapeutic strategy for premenopausal women with hormone-receptor-positive (HR+) breast cancer and those who experience chemotherapy-associated amenorrhea similarly experience improved risk of recurrence^6,7^. Recent data from Suppression of Ovarian Function Trial (SOFT) and Tamoxifen vs Exemestane Trial (TEXT) have identified substantial improvements in survival outcomes with the addition of OFS to tamoxifen, with particular benefit among women age <35^6,8^. However, chemotherapy-related amenorrhea and OFS may have many negative impacts, including infertility or subfertility, vasomotor symptoms, impaired sexual functioning, and long-term medical sequelae including bone loss^9^.

The ability to predict which patients will experience transient or permanent loss of menstrual function is important in counseling patients on the risks of chemotherapy, potential benefits of fertility preservation, and the management of ovarian function during adjuvant endocrine therapy. The vast majority of studies examining rates of treatment-related amenorrhea (TRA) were completed prior to the use of modern regimens, including docetaxel/cyclophosphamide (TC) and docetaxel/carboplatin/trastuzumab (TCH), and there are limited data regarding TRA following dose-dense doxorubicin/cyclophosphamide (AC) regimens^7,10–13^. Here we describe patient-reported menstrual function outcomes, including development of TRA and resumption of menses from a large, prospective cohort of young women with early breast cancer.

## Results

### Patient characteristics

Among 954 eligible participants, 789 women were evaluable at the 1-year timepoint, among whom 109 (13.8%) were at age ≤30 at diagnosis, 216 (27.4%) at age 31–35, and 464 (58.8%) at age 36–40, with a median age of 36. The majority (592/782, 75.0%) received chemotherapy (Table 1), including 16 women who received less than 50% of the standard total dose of an agent. No dose reductions for obesity were identified. Patient, disease, and treatment factors for patients excluded because of missing surveys at baseline/6-month surveys or 1-year surveys are described in Supplementary Table 1.

**Table 1:** Patient, disease, and treatment characteristics.

Age at diagnosis (years)	*n* (%)
≤30	109 (13.8)
31–35	216 (27.4)
36–40	464 (58.8)
BMI at diagnosis	
<18.5	38 (4.8)
18.5–24.9	476 (60.3)
≥25	241 (30.6)
Missing	34 (4.3)
Race	
Non-White	118 (15.0)
White	671 (85.0)
Smoking	
Active/former	255 (32.3)
Never	492 (62.4)
Missing	42 (5.3)
Stage	
0	67 (8.5)
I	275 (34.8)
II	340 (43.1)
III	107 (13.6)
HR expression	
No	186 (23.2)
Yes	602 (76.3)
Missing	1 (0.5)
HER2 amplification	
No	505 (64.0)
Yes	229 (29.0)
Indeterminate/missing	55 (7.0)
Tamoxifen use at 1 year	
No	296 (37.5)
Yes	459 (58.2)
Missing	34 (4.3)
Chemotherapy	
Yes	592 (75.0)
No	197 (25.0)

*BMI* body mass index, *HR* hormone receptor, *HER2* human epidermal growth factor receptor 2.

### Menstrual patterns

The proportions of evaluable participants with active menstrual function, defined as having a period within the prior 12 months, at each annual timepoint within the first five years following diagnosis were: 1 year: 710/789 (90.0%); 2 year: 537/699 (76.8%); 3 year: 444/592 (75.0%); 4 year: 391/511 (76.5%); 5 year: 314/430 (73.0%). To acknowledge that some women experience bleeding in the first few months after initiating treatment before ovarian function is impacted, we evaluated TRA at 1 year as the proportion with no period within 6 months prior (286/789; 36.2%) and at 2 years as the proportion with no period within the 18 months prior (120/699; 17.2%).

Among the participants who had TRA at the 1-year and 2-year timepoints, 183/286 (63.9%) and 13/120 (10.8%), respectively, had resumption of menstrual function reported on a subsequent survey within 5 years following diagnosis.

Of the participants who had a last menstrual period (LMP) within 12 months of the 2-year, 3-year, and 4-year timepoints, the proportions who became amenorrheic at the subsequent timepoints were: 2 year: 43/537 (8.0%); 3 year: 27/444 (6.1%); 4 year: 18/391 (4.6%).

### Patient and treatment characteristics associated with TRA

The proportions of participants at age ≤30, 31–35, and 36–40 who experienced TRA at 1 year were 23/109 (21.1%), 140/216 (35.2%), and 187/464 (40.3%) and at 2 years were 10/82 (12.2%), 25/149 (16.8%), and 53/307 (17.3%), respectively. Among 80 participants across all age groups who received neither tamoxifen nor chemotherapy, 5 (6.3%) experienced 1-year TRA. Among 115 participants who received tamoxifen but no chemotherapy, 23 (20.0%) experienced 1-year TRA. Of 592 participants who received chemotherapy, 258 (43.6%) experienced 1-year TRA vs 28/197 (14.2%) of those who did not receive chemotherapy.

**Fig. 1:**
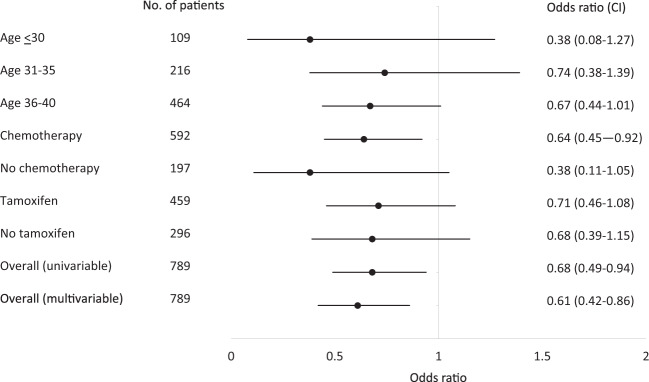
Forest plot analyses to evaluate the association between BMI ≥ 25 (vs BMI 18.5–24.9) and 1-year treatment-related amenorrhea are shown. A consistent association between BMI ≥ 25 and a lower odds of 1-year TRA was present among each age group and by receipt or non-receipt of chemotherapy and tamoxifen.

Characteristics associated with 1-year TRA in univariable analysis included older age, normal body mass index (BMI) (compared to higher), chemotherapy use, and tamoxifen use, whereas race and smoking were not (Table 2). In the multivariable logistic regression model, the following characteristics remained associated with TRA: age (OR_≤30 vs 36-40_ = 0.29 (0.17–0.48), OR_31-35 vs 36-40_ = 0.67 (0.46–0.94), *p* = 0.02); BMI (OR_≥25 vs 18.5-24.9_ = 0.59 (0.41–0.83), *p* < 0.01; OR_<18.5 vs 18.5-24.9_ = 0.90 (0.41–1.89), *p* = 0.78); chemotherapy use (vs not; OR = 5.55 (3.60–8.82), *p* < 0.01; and tamoxifen use (vs not; OR = 1.55 (1.11–2.16), *p* = 0.01). There appeared to be a consistent association between BMI ≥ 25 and a lower odds of 1-year TRA among each age group and by receipt or non-receipt of chemotherapy and tamoxifen (Fig. 1). To further explore the association between BMI and 1-year TRA, participants with a BMI ≥ 25 were divided into overweight (BMI 25.0–29.9) (*n* = 155) and obese (BMI ≥ 30) (*n* = 86) in a univariable model. Rates of 1-year TRA were 45/155 (29.0%) and 28/86 (32.6%), respectively, and the direction and magnitude of the associations with TRA were similar (OR_25-29.9 vs 18.5-24.9_ = 0.64 (0.43–0.94), *p* = 0.025; OR_≥30 vs 18.5-24.9_ = 0.75 (0.46–1.21), *p* = 0.25), though not statistically significant in the obese (vs normal BMI) patients.

**Table 2:** Proportion of patients with 1-year TRA and associated characteristics among 789 eligible participants.

Characteristic	TRA at 1 year	Univariable analysis	*p*-value	Multivariable analysis	*p*-value
	*n* (%)	OR (95% CI)		OR (95% CI)	
Total cohort	286/789 (36.2%)				
Age at diagnosis (years)					
≤30	23/109 (21.1%)	0.40 (0.24–0.64)	<0.01	0.29 (0.17–0.48)	<0.001
31–35	76/216 (35.2%)	0.80 (0.57–1.12)	0.20	0.66 (0.46–0.94)	0.024
36–40	187/464 (40.3%)	Ref	Ref	Ref	Ref
BMI					
<18.5	12/38 (31.6%)	0.72 (0.34–1.43)	0.36	0.90 (41–1.90)	0.792
18.5–24.9	186/476 (39.1%)	Ref	Ref	Ref	Ref
≥25	73/241 (30.3%)	0.68 (0.49–0.94)	0.02	0.61 (0.42–0.86)	0.006
Missing	15/34 (44.1%)				
Race					
Non-White	43/118 (36.4%)	1.00 (0.67–1.51)	0.96		
White	243/671 (36.2%)	Ref	Ref		
Smoking					
Active/former	83/255 (32.6%)	0.79 (0.58–1.09)	0.16	0.83 (0.59–1.17)	0.296
Never	186/492 (37.8%)	Ref		Ref	Ref
Missing	17/42 (40.5%)				
Tamoxifen use at 1 year					
No	89/296 (30.1%)	Ref	Ref	Ref	Ref
Yes	182/459 (39.7%)	1.53 (1.12–2.09)	<0.01	1.55 (1.12–2.16)	0.009
Missing	15/34 (44.1%)				
Chemotherapy					
Yes	258/592 (43.6%)	4.66 (3.07–7.31)	<0.01	5.57 (3.62–8.86)	<0.001
No	28/197 (14.2%)	Ref	Ref	Ref	Ref

*CI* confidence interval, *OR* odds ratio, *TRA* treatment-related amenorrhea.

The raw proportions of participants who experienced 1-year TRA and 2-year TRA by age and the most common chemotherapy regimens are reported in Tables 3 and 4 and for less common regimens in Supplementary Table 2. Overall, 1-year TRA rates associated with AC-paclitaxel (AC-T) (primarily administered dose dense), TC, and TCH regimens were similar (44.1%, 41.8%, and 55.6%, respectively), but the TRA rate was lower with AC (25%) and even lower with weekly paclitaxel and trastuzumab (11.1%). Among women receiving AC-T, the most commonly used regimen, rates of 1-year TRA among women at age ≤30, 31–35, and 36–40 were 11/59 (18.6%), 43/105 (41.0%), and 103/192 (53.6%), respectively.

**Table 3:** Rates of 1-year treatment-related amenorrhea following common adjuvant chemotherapy regimens and overall by age.

Regimen	Age at diagnosis (years)			
	≤30	31–35	36–40	Overall
AC (+/−H, +/−P)	2/3 (66.7%)	2/15 (13.3%)	6/22 (27.3%)	10/40 (25.0%)
AC-T(+/−H, +/−P)	11/59 (18.6%)	43/105 (41.0%)	103/192 (53.6%)	157/356 (44.1%)
TC (+/−H, +/−P)	5/18 (27.8%)	7/16 (43.8%)	16/33 (48.5%)	28/67 (41.8%)
TH	0/5 (0%)	0/2 (0%)	3/20 (15.0%)	3/27 (11.1%)
TCH (+/−P)	1/2 (50.0%)	6/15 (40.0%)	13/19 (68.4%)	20/36 (55.6%)
All regimens	21/93 (22.6%)	68/190 (35.8%)	169/329 (51.4%)	258/592 (43.6%)

AC (doxorubicin, cyclophosphamide); H (trastuzumab); P (pertuzumab); AC-T (doxorubicin, cyclophosphamide, paclitaxel); TC (docetaxel, cyclophosphamide); TH (weekly paclitaxel and trastuzumab); TCH (docetaxel, carboplatin, trastuzumab).

**Table 4:** Rates of 2-year treatment-related amenorrhea following common adjuvant chemotherapy regimens and overall by age.

Regimen	Age at diagnosis (years)			
	≤30	31–35	36–40	Overall
AC (+/−H, +/−P)	1/4 (25.0%)	1/14 (7.1%)	3/120 (2.5%)	5/138 (3.6%)
AC-T(+/−H, +/−P)	5/60 (8.3%)	19/97 (19.6%)	43/172 (25%)	67/329 (20.4%)
TC (+/−H, +/−P)	2/17 (11.8%)	2/17 (11.8%)	5/24 (20.8%)	9/58 (15.5%)
TH	0/5 (0%)	1/3 (33.3%)	2/19 (10.5%)	3/27 (11.1%)
TCH (+/−P)	0/1 (0%)	1/14 (7.1%)	3/17 (17.6%)	4/32 (12.5%)
All regimens	10/93 (10.8%)	27/160 (16.9%)	65/286 (22.7%)	102/539 (18.9%)

AC (doxorubicin, cyclophosphamide); H (trastuzumab); P (pertuzumab); AC-T (doxorubicin, cyclophosphamide, paclitaxel); TC (docetaxel, cyclophosphamide); TH (weekly paclitaxel and trastuzumab); TCH (docetaxel, carboplatin, trastuzumab).

## Discussion

Over the past several years, the critical implications of ovarian function on the management and outcomes of young women with breast cancer have become well-recognized. In NSABP B30, amenorrhea lasting 6 months or longer following chemotherapy was associated with improved disease-free and overall survival^7^. Similarly, the addition of OFS to tamoxifen also improves disease-free and overall survival^6,14^. On the other hand, loss of ovarian function increases the risk of menopausal symptoms and medical comorbidities and results in infertility^9^. The ability to accurately predict which young patients will experience temporary or permanent amenorrhea, and which patients will not, is needed upon diagnosis and in follow-up to inform the optimal treatment plan. Our data demonstrate that, in a large prospective cohort of young women treated with modern chemotherapy regimens, short episodes of TRA are common, but the vast majority will maintain or regain menstrual function.

The ability to predict loss of ovarian function may be most critical in informing the decision to use fertility preservation, including embryo and oocyte cryopreservation. While TRA is an imperfect surrogate of future fertility—those who regain menstrual function may still be infertile or subfertile—and any risk of treatment-related infertility may be enough to warrant fertility preservation for some women, our data help inform which patients may be at very low risk of TRA and, therefore, less likely to need fertility preservation. For instance, only 20% of women at age 30 or younger experienced even a short course of amenorrhea. Similarly, very low rates of TRA were reported following paclitaxel/trastuzumab, which omits alkylating chemotherapy, the class most strongly associated with gonadotoxicity. Therefore, these patients may be less likely to need banked embryos or oocytes to conceive in the future, though additional research is needed to better stratify risk of infertility and pre-treatment biomarkers such as AMH are being explored. Rates of 1-year TRA were more moderate for patients at ages 31–35 and 36–40, yet the majority had subsequent resumption of menstrual function by the 2-year timepoint, supporting the 2-year cutoff for defining TRA^15^. While fertility preservation is important for many women, it may not be feasible for many others due to cost, availability, or the need to urgently initiate treatment for symptomatic disease. Additionally, while controlled ovarian stimulation for fertility preservation appears safe^16,17^, the available data are somewhat limited, particularly for patients receiving neoadjuvant chemotherapy^18,19^ and for whom delays in care could be more impactful than those who undergo fertility preservation following resection of the tumor. For young women who face such barriers to fertility preservation use and are at low risk of TRA, our finding that they are likely to maintain or regain ovarian function in the years following diagnosis may be reassuring.

Another common treatment decision for young breast cancer patients completing chemotherapy for HR+ breast cancer is whether and when to initiate therapeutic OFS. These data support initiation of OFS prior to resumption of menses following chemotherapy for women at age 40 or younger, given that fewer than 20% of women at age 35–40 had TRA 2 years following diagnosis. This approach prevents women from undergoing a second menopausal transition with initiation of OFS following resumption of menses. Additionally, this approach addresses the common challenge of how to incorporate use of aromatase inhibitors (AI) for young women following chemotherapy, due to the concern that resumption of menstrual function would interrupt effective endocrine therapy if an AI alone were given in this setting. Recognizing that the routine use of GnRH agonist in this setting would lead to overtreatment of a small proportion of young women who do experience permanent menopause, this decision should be tailored to the individual patient’s risk of long-term amenorrhea, breast cancer risk features, and concerns regarding side effects, out-of-pocket costs, and the burden of injection visits.

Our data demonstrate similar rates of TRA across the most frequently used modern multi-agent chemotherapy regimens AC-T +/− H +/− P, TC +/− H +/− P, and TCH +/− P. The number of important confounders (age, tamoxifen use, BMI) and variety of chemotherapy regimens precludes the evaluation of an association between specific regimens and TRA. However, the higher numerical rate of TRA associated with AC-T relative to AC is consistent with prior studies demonstrating that taxanes contribute to amenorrhea^11,20–22^. As noted previously, the rate of TRA among patients receiving weekly paclitaxel/trastuzumab was low, consistent with prior studies^23^. Only very limited data are available describing TRA associated with use of platinum chemotherapy for young breast cancer patients, and in other settings as well. In a small retrospective study, the rate of TRA following carboplatin/taxane chemotherapy was only 13%^24^. On the other hand, an analysis of the ALTTO trial found that the TRA rate was higher following TCH than anthracycline-taxane chemotherapy, though comparisons may have been limited by differences in the taxanes used^21^. In the Childhood Cancer Survivor Study, one of the largest studies to evaluate the impact of chemotherapy on menstrual function, albeit among children rather than adults, cisplatin use was not associated with long-term fertility outcomes^25^. The rate of TRA associated with docetaxel-carboplatin-trastuzumab (TCH) in this study was numerically higher than that of patients treated with docetaxel-cyclophosphamide. While these data are unadjusted and there are differences in standard number of cycles (6 vs 4, respectively), cyclophosphamide is generally regarded as the main driver of TRA among breast cancer patients and the high rates of TRA associated with TCH suggest that carboplatin does contribute to TRA and warrant further attention in future studies.

Our finding that overweight and obese women treated with chemotherapy in this study experienced a 40% lower risk of TRA than those with a normal body weight is striking. The association between overweight/obesity and a lower odds of TRA appeared consistent across subpopulations. In particular, the association seemed similar among women who did vs did not receive chemotherapy, suggesting that the lower odds of TRA among overweight/obese women is not solely mediated by differences in chemotherapy pharmacokinetics by body mass. Most studies evaluating TRA among young women have found no association between BMI and TRA^20,21,23,26^. A prior prospective study of young women found that overweight women were more likely to experience amenorrheic episodes, but no association was identified between obesity and TRA^11^. Our study is the largest to prospectively evaluate menstrual function among breast cancer patients, potentially contributing to the ability to detect an association that was obscured by prior smaller studies. In the POSH study, obese women at age ≤40 years with estrogen-receptor-positive (ER+) breast cancer experienced significantly inferior distant disease-free interval and overall survival relative to those with normal weights^27^. Only a small proportion of patients had received OFS and the analysis did not adjust for TRA. While the relationship between obesity and breast cancer recurrence risk is complex, the consistent association between amenorrhea and improved breast cancer outcomes^7,21,28^ raises the question of whether the lower risk of TRA observed might contribute to the inferior outcomes experienced by obese patients. Even among young women with HR+ breast cancer treated with OFS with a GnRH agonist, obese patients are more likely to experience elevated estradiol levels, and incomplete OFS may underlie a higher recurrence risk in this setting as well^29,30^. Obese postmenopausal women with ER+ breast cancer treated with AIs in the adjuvant setting also experience inferior breast cancer outcomes, possibly due to inadequate suppression of aromatase, which is produced in adipose tissue^31–33^ and this has been observed among premenopausal women as well^33^. These accumulating data have implications for future research and patient care, especially as the proportion of women in many populations who are overweight or obese is increasing.

This study should be interpreted in the context of its limitations. Some subgroups by age, BMI, and chemotherapy regimen contain small numbers of participants, limiting comparisons and the ability to incorporate specific subgroups into the multivariable analysis. Menstrual function and BMI were evaluated by patient report and potentially subject to recall and other biases. The proportion of participants with BMI ≥ 25 may have been higher among those excluded due to missing 1-year survey information, though TRA data were not available for these patients, limiting analysis of how this could impact the findings. While TRA is an important surrogate for ovarian function, some women may have residual ovarian estradiol production without menstruating. Future analyses will evaluate serum FSH and estradiol levels at serial timepoints to further examine menstrual function among young women treated for early breast cancer. Additionally, TRA is also an imperfect surrogate for ovarian reserve damage and fertility and additional analyses are warranted to evaluate pregnancy and birth outcomes.

In conclusion, the management of young women with breast cancer should be informed by accurate information regarding the risk of TRA with modern treatment to optimize disease outcomes and preserve fertility when desired. Women at age ≤40 diagnosed with breast cancer experience moderate rates of early TRA but most will resume menstrual function within 2 years and this varies by patient factors including age and body mass. Future research to confirm and further understand the relationship between BMI, TRA, and breast cancer outcomes as well as to incorporate novel biomarkers to improve the prediction of TRA for young patients is clearly warranted.

## Methods

### Study design

The Young Women’s Breast Cancer Study (YWS) is a multi-center, prospective cohort study of women diagnosed with breast cancer at age ≤40. Participants were enrolled from 12 sites in the United States and Canada from 2006 to 2016 within 6 months of diagnosis. Those who were able to respond to questionnaires in English were eligible. Potential participants at Dana-Farber/Harvard Cancer Center (DF/HCC) sites were identified by the Rapid Case Identification Core through pathology record review and elsewhere through systematic review of clinic lists. Women who did not return the baseline survey during the recruitment period and neither consented nor declined participation were invited 1 year after diagnosis to participate with abbreviated “short form” surveys. Participants at Sunnybrook Health Sciences Centre completed abbreviated surveys at all timepoints. Follow-up surveys were sent every 6 months for 3 years (with the third survey timed to be answered approximately 1 year from diagnosis and subsequent survey timepoints based on that reset timing) and are sent annually thereafter. Participants provided written informed consent, authorizing medical record review, participant questionnaires, and biospecimen collection. IRB approval for the study was obtained through DF/HCC and other participating centers.

### Study population

**Fig. 2:**
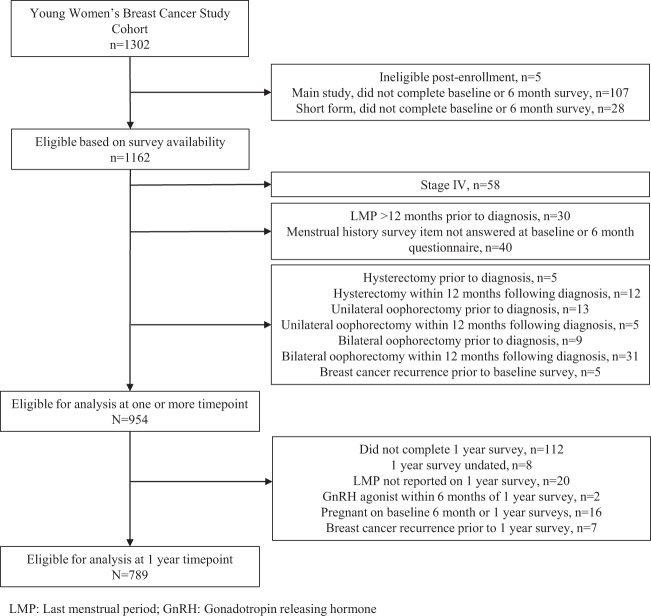
Participant flow diagram. 789 eligible participants were evaluable at the 1-year timepoint.

This analysis describes patient-reported menstrual outcomes for participants with stage 0–III breast cancer (Fig. 2). Participants who did not complete surveys administered at baseline or 6 months after diagnosis (*n* = 135), or the menstrual history question on these surveys (*n* = 40), were excluded, as were participants who reported having undergone hysterectomy (*n* = 17), unilateral oophorectomy (*n* = 18), or bilateral oophorectomy (*n* = 40) at baseline or 6 months. Participants who reported a LMP more than 1 year prior to diagnosis (*n* = 30) were classified as postmenopausal and excluded. Participants with stage IV disease at diagnosis (*n* = 58) or with recurrence within 1 year (*n* = 5) were excluded given substantial differences in treatment patterns. Participants who later reported being pregnant or receiving a gonadotropin receptor hormone (GnRH) agonist within 1 year were censored at those timepoints but eligible at subsequent timepoints. Participants who later underwent a hysterectomy, unilateral oophorectomy, or bilateral oophorectomy, or experienced a disease recurrence were censored at all subsequent timepoints.

### Outcomes

We evaluated menstrual outcomes in the 5 years following diagnosis. The proportion with active menstrual function, defined as the number of participants with LMP within 1 year of completing the survey over the number who were evaluable, was calculated at annual timepoints. TRA at 1 year was defined as the number of eligible participants whose LMP was greater than 6 months prior to completing the survey, and TRA at 2 years as the proportion whose LMP was greater than 18 months prior to completing the survey, to recognize that amenorrhea may not occur immediately upon treatment initiation and, therefore, TRA should not be defined using menstrual function in the first 6 months after diagnosis. Resumption of menses was defined as the proportion of women with TRA at 1 and 2 years who reported a period on a subsequent survey through the 5-year timepoint. We also assessed the development of amenorrhea, defined as having one or more amenorrheic periods of 1 year or longer, among women who did not have TRA at 1 year.

### Other covariates

Disease and treatment information, including stage, HR expression, and chemotherapy use was obtained through medical record review, including each dose of chemotherapy and doses reduced, held, or delayed. Race, smoking status, and BMI were self-reported on the baseline survey. Chemotherapy regimens were categorized based upon the entirety of treatment received for (neo)adjuvant therapy. Receipt of trastuzumab and/or pertuzumab vs not was collapsed within the cytotoxic chemotherapy regimen given prior data finding no impact of trastuzumab on TRA^20^. Dose reduction >50% was characterized as receipt of any drug at 50% or less of the standard total dose, including held and reduced doses. Endocrine therapy use, including oral endocrine therapies and GnRH agonists, at serial timepoints was obtained from completed surveys and tamoxifen use at 1 year is reported.

### Statistical analysis

We identified patient and treatment characteristics potentially associated with TRA. Those which were significantly associated with 1-year TRA in univariable analysis (*p* < 0.20) were evaluated in a multivariable logistic regression model^34^. To evaluate the association between BMI and TRA and interactions with other relevant patient and treatment factors, univariable analyses were performed within relevant subpopulations.

### Reporting summary

Further information on research design is available in the Nature Research Reporting Summary linked to this article.

## Supplementary information

Supplementary information.

Reporting summary.

## Data Availability

The data generated and analyzed during this study are described in the following data record: 10.6084/m9.figshare.14828298^35^. The data underlying the final analyses are contained in 52.csv files. These files are not publicly available as the IRB-approved research protocol specified that all data must be collected, coded, and stored at the Dana-Farber Cancer Institute and be limited-access and password-protected in the Partners system, in order to protect the identity of respondents. Requests can be made to share data privately. However, any data sharing will require a formal data transfer agreement between the Dana-Farber Cancer Institute and the other party. Requests to this effect should be directed to the corresponding author.
