# Supplementary material for: Treatment-related amenorrhea in a modern, prospective cohort study of young women with breast cancer
Source: NPJ Breast Cancer. 2021 Jul 27;7:99. doi: 10.1038/s41523-021-00307-8 (PMC8316568; doi:10.1038/s41523-021-00307-8)
Supplement: Supplementary file 1 — Supplementary information. [file 41523_2021_307_MOESM1_ESM.pdf]

SUPPLEMENTARY MATERIAL

Supplementary Table 1: Patient, disease, and treatment factors for patients excluded because of missing surveys at baseline/6-month surveys or 1-year surveys

Age at Diagnosis	Missing baseline and 6-month survey N (%)	Missing 1-year survey N (%)
≤30	12 (8.9)	26 (15.8%)
31-35	33 (24.4)	53 (32.1%)
36-40	90 (66.7)	86 (52.1%)
BMI at diagnosis		
<18.5	0	4 (2.4%)
18.5-24.9	0	67 (40.6%)
≥25	0	56 (33.9%)
Missing	135 (100)	38 (23.0%)
Race		
Non-White	39 (28.1)	38 (23.0%)
White	96 (71.1)	127 (77.0%)
Smoking		
Active/Former	0	41 (24.8%)
Never	0	87 (52.7%)
Missing	135 (100)	37 (22.4%)
Stage		
0	8 (5.9)	9 (5.5)
I	36 (26.7)	50 (30.3)
II	57 (42.2)	71 (43.0)
III	28 (20.7)	35 (21.2)
IV	6 (4.4)	0 (0)
HR expression		
No	33 (24.4)	41 (24.8)
Yes	102 (75.6)	124 (75.2)
Missing	0	
HER2 amplification		
No	92 (68.1)	115 (69.7)
Yes	34 (25.2)	45 (27.3)
Indeterminate/missing	9 (6.7)	5 (3.0)
Tamoxifen use at 1 year		
No	12 (8.9)	34 (20.6)
Yes	7 (5.2)	28 (17.0)
Missing	116 (85.9)	103 (62.4)

Chemotherapy		
Yes	90 (66.7)	131 (79.4%)
No	45 (33.3)	34 (20.6%)

BMI: body mass index; HR: hormone receptor; HER2: human epidermal growth factor receptor 2

Supplementary Table 2: Rates of 1-year treatment-related amenorrhea by each chemotherapy regimen and age group

Regimen	Age at diagnosis (years)			
	≤30	31-35	36-40	Total
AC (+/-H, +/-P)	2/3 (66.7%)	2/15 (13.3%)	6/22 (27.3%)	10/40 (25.0%)
AC-T(+/-H, +/-P)	11/59 (18.6%)	43/105 (41.0%)	103/192 (53.6%)	157/356 (44.1%)
AC-D (+/H, +/-P)	2/2 (100%)	4/7 (57.1%)	8/10 (80%)	14/19 (73.7%)
TC (+/- H, +/-P)	5/18 (27.8%)	7/16 (43.8%)	16/33 (48.5%)	28/67 (41.8%)
TH	0/5 (0%)	0/2 (0%)	3/20 (15.0%)	3/27 (11.1%)
TCH (+/-P)	1/2 (50.0%)	6/15 (40.0%)	13/19 (68.4%)	20/36 (55.6%)
Paclitaxel/Carboplatin	N/A	1/1 (100%)	NA	1/1 (100%)
AC-T + Carboplatin	NA	0/1 (0%)	4/6 (66.7%)	4/7 (57.1%)
AC-D + Carboplatin	0/1 (0%)	NA	2/2 (100%)	2/3 (66.7%)
AC-T + Cisplatin	0/1 (0%)	2/3 (66.7%)	4/6 (66.7%)	6/10 (60.0%)
AC + Cisplatin	NA	NA	3/4 (75.0%)	3/4 (75.0%)
FEC-D	0/1 (0%)	1/1 (100%)	4/6 (66.7%)	5/8 (62.5%)
FEC-T	NA	0/1 (100%)	1/1 (100%)	1/2 (50.0%)
Non-standard regimen	0/1 (0%)	2/3 (66.7%)	2/8 (25.0%)	4/12 (33.3%)
All regimens	21/93 (22.6%)	68/190 (35.8%)	169/329 (51.4%)	258/592 (43.6%)

AC (doxorubicin, cyclophosphamide); H (trastuzumab); P (pertuzumab); AC-T (doxorubicin, cyclophosphamide, paclitaxel); AC-D (doxorubicin, cyclophosphamide, docetaxel); TC (docetaxel, cyclophosphamide); TH (weekly paclitaxel and trastuzumab); TCH (docetaxel, carboplatin, trastuzumab); FEC-D (fluorouracil, epirubicin, cyclophosphamide, docetaxel); FEC-T (fluorouracil, epirubicin, cyclophosphamide, paclitaxel); NA (empty cell)
