# Supplementary material for: Treatment-related amenorrhea in a modern, prospective cohort study of young women with breast cancer
Source: NPJ Breast Cancer. 2021 Jul 27;7:99. doi: 10.1038/s41523-021-00307-8 (PMC8316568; doi:10.1038/s41523-021-00307-8)
Supplement: Supplementary file 2 — Reporting summary. [file 41523_2021_307_MOESM2_ESM.pdf]

nature research

Corresponding author(s): Ann Partridge

Last updated by author(s): Jun 18, 2021

Reporting Summary

Nature Research wishes to improve the reproducibility of the work that we publish. This form provides structure for consistency and transparency in reporting. For further information on Nature Research policies, see our Editorial Policies and the Editorial Policy Checklist.

Statistics

For all statistical analyses, confirm that the following items are present in the figure legend, table legend, main text, or Methods section.

n/a	Confirmed	
☐	☒	The exact sample size (*n*) for each experimental group/condition, given as a discrete number and unit of measurement
☒	☐	A statement on whether measurements were taken from distinct samples or whether the same sample was measured repeatedly
☐	☒	The statistical test(s) used AND whether they are one- or two-sided<br>*Only common tests should be described solely by name; describe more complex techniques in the Methods section.*
☐	☒	A description of all covariates tested
☐	☒	A description of any assumptions or corrections, such as tests of normality and adjustment for multiple comparisons
☐	☒	A full description of the statistical parameters including central tendency (e.g. means) or other basic estimates (e.g. regression coefficient) AND variation (e.g. standard deviation) or associated estimates of uncertainty (e.g. confidence intervals)
☐	☒	For null hypothesis testing, the test statistic (e.g. *F*, *t*, *r*) with confidence intervals, effect sizes, degrees of freedom and *P* value noted<br>*Give P values as exact values whenever suitable.*
☒	☐	For Bayesian analysis, information on the choice of priors and Markov chain Monte Carlo settings
☒	☐	For hierarchical and complex designs, identification of the appropriate level for tests and full reporting of outcomes
☐	☒	Estimates of effect sizes (e.g. Cohen's *d*, Pearson's *r*), indicating how they were calculated

Our web collection on statistics for biologists contains articles on many of the points above.

Software and code

Policy information about availability of computer code

Data collection	Data collection was performed using Microsoft Access and REDCap software.
Data analysis	Data analysis was performed using SAS Version 9.4 (Cary, NC). Statistical analyses are described in detail. Code is not available to protect participant privacy.

For manuscripts utilizing custom algorithms or software that are central to the research but not yet described in published literature, software must be made available to editors and reviewers. We strongly encourage code deposition in a community repository (e.g. GitHub). See the Nature Research guidelines for submitting code & software for further information.

Data

Policy information about availability of data

All manuscripts must include a data availability statement. This statement should provide the following information, where applicable:

- Accession codes, unique identifiers, or web links for publicly available datasets
- A list of figures that have associated raw data
- A description of any restrictions on data availability

The data are not publicly available due to study restrictions which preclude sharing of data to protect participant privacy. Qualified researchers can contact the corresponding author (ann_partridge@dfci.harvard.edu) to apply for access to the datasets.

Field-specific reporting

Please select the one below that is the best fit for your research. If you are not sure, read the appropriate sections before making your selection.

☒ Life sciences ☐ Behavioural & social sciences ☐ Ecological, evolutionary & environmental sciences

For a reference copy of the document with all sections, see nature.com/documents/nr-reporting-summary-flat.pdf

Life sciences study design

All studies must disclose on these points even when the disclosure is negative.

Sample size	This is a retrospective analysis of a prospective cohort. The sample size of the prospective cohort was determined a prior for different analyses.
Data exclusions	This analysis describes patient-reported menstrual outcomes for participants with stage 0-III breast cancer. Participants who did not complete surveys administered at baseline or 6 months after diagnosis (n=135), or the menstrual history question on these surveys (n=40), were excluded, as were participants who reported having undergone hysterectomy (n=17), unilateral oophorectomy (n=18), or bilateral oophorectomy (n=40) at baseline or 6-months. Participants who reported a last menstrual period (LMP) more than one year prior to diagnosis (n=30) were classified as postmenopausal and excluded. Participants with stage IV disease at diagnosis (n=58) or with recurrence within 1 year (n=5) were excluded given substantial differences in treatment patterns. Participants who later reported being pregnant or receiving a gonadotropin receptor hormone (GnRH) agonist within 1 year were censored at those timepoints but eligible at subsequent timepoints. Participants who later underwent a hysterectomy, unilateral oophorectomy, or bilateral oophorectomy, or experienced a disease recurrence were censored at all subsequent timepoints.
Replication	Models were verified by multiple members of the study team, including clinical investigators and multiple statisticians.
Randomization	This is a prospective cohort study. Participants were not randomized.
Blinding	No blinding was performed.

Reporting for specific materials, systems and methods

We require information from authors about some types of materials, experimental systems and methods used in many studies. Here, indicate whether each material, system or method listed is relevant to your study. If you are not sure if a list item applies to your research, read the appropriate section before selecting a response.

Materials & experimental systems

n/a	Involved in the study	
☒	☐	Antibodies
☒	☐	Eukaryotic cell lines
☒	☐	Palaeontology and archaeology
☒	☐	Animals and other organisms
☐	☒	Human research participants
☐	☒	Clinical data
☒	☐	Dual use research of concern

Methods

n/a	Involved in the study	
☒	☐	ChIP-seq
☒	☐	Flow cytometry
☒	☐	MRI-based neuroimaging

Human research participants

Policy information about studies involving human research participants

Population characteristics	The Young Women's Breast Cancer Study (YWS) is a multi-center, prospective cohort study of women diagnosed with breast cancer at age <40. Participants were enrolled from 12 sites in the United States and Canada from 2006-2016 within six months of diagnosis. Those who were able to respond to questionnaires in English were eligible.
Recruitment	Potential participants at Dana-Farber/Harvard Cancer Center (DF/HCC) sites were identified by the Rapid Case Identification Core through pathology record review and elsewhere through systematic review of clinic lists.
Ethics oversight	IRB approval for the study was obtained through DF/HCC and other participating centers.

Note that full information on the approval of the study protocol must also be provided in the manuscript.

Clinical data

Policy information about clinical studies

All manuscripts should comply with the ICMJE guidelines for publication of clinical research and a completed CONSORT checklist must be included with all submissions.

Clinical trial registration	NCT01468246
Study protocol	The study protocol has not been published. Relevant inquiries can be made to the corresponding author.
Data collection	Disease and treatment information, including stage, HR expression, and chemotherapy use was obtained through medical record review, including each dose of chemotherapy and doses reduced, held, or delayed. Race, smoking status, and body mass index (BMI) were self-reported on the baseline survey. Menstrual history data was self-reported on baseline and subsequent surveys.
Outcomes	We evaluated menstrual outcomes in the five years following diagnosis. The proportion with active menstrual function, defined as the number of participants with LMP within one year of completing the survey over the number who were evaluable, was calculated at annual timepoints. TRA at 1-year was defined as the number of eligible participants whose LMP was greater than 6 months prior to completing the survey, and TRA at 2 years as the proportion whose LMP was greater than 18 months prior to completing the survey, to recognize that amenorrhea may not occur immediately upon treatment initiation and, therefore, TRA should not be defined using menstrual function in the first six months after diagnosis. Resumption of menses was defined as the proportion of women with TRA at 1 and 2 years who reported a period on a subsequent survey through the five-year timepoint. We also assessed the development of amenorrhea, defined as having one or more amenorrheic periods of one year or longer, among women who did not have TRA at 1 year.
